# Perspectives From Municipality Officials on the Adoption, Dissemination, and Implementation of Electronic Health Interventions to Support Caregivers of People With Dementia: Inductive Thematic Analysis

**DOI:** 10.2196/17255

**Published:** 2020-05-13

**Authors:** Hannah Liane Christie, Mignon Chloë Philomela Schichel, Huibert Johannes Tange, Marja Yvonne Veenstra, Frans Rochus Josef Verhey, Marjolein Elizabeth de Vugt

**Affiliations:** 1 Department of Psychiatry and Neuropsychology and Alzheimer Centre Limburg School for Mental Health and Neurosciences Maastricht University Maastricht Netherlands; 2 CAPHRI School for Public Health and Primary Care Maastricht Netherlands; 3 Department of Family Practice, CAPHRI School for Public Health and Primary Care Maastricht University Maastricht Netherlands; 4 Burgerkracht Limburg Sittard Netherlands

**Keywords:** dementia, caregiver, internet, eHealth, implementation, senior friendly communities

## Abstract

**Background:**

Very few evidence-based electronic health (eHealth) interventions for caregivers of people with dementia are implemented into practice. As part of a cross-border collaboration focusing on dementia and depression in older people, two eHealth interventions for caregivers of people with dementia (“Myinlife” and “Partner in Balance”) were adopted by nine municipalities in the Euregion Meuse-Rhine.

**Objective:**

This study aimed to (1) identify determinants for the implementation of eHealth interventions for caregivers of people with dementia in a municipality context and (2) formulate implementation strategies for these interventions.

**Methods:**

Eight municipality officials were interviewed using open-ended, semistructured interviews about their background, thoughts on the implementation of the intervention, recommended strategies, and thoughts on eHealth in general. One additional municipality discontinued the implementation project and submitted answers to the interview questions via email. The interviews were transcribed and independently analyzed using inductive thematic analysis.

**Results:**

The interviews provided information on the perspectives of municipality officials on implementing eHealth for caregivers of people with dementia in their local communities. Key findings from the inductive thematic analysis included the importance of face-to-face interviews in developing tailor-made implementation plans, the need for regular meetings, the enthusiasm of municipality officials to implement these interventions, the need for long-term sustainability planning through collecting data on the required resources and benefits, and the effect of name brand recognition in adoption.

**Conclusions:**

The findings contribute toward filling the previously identified gap in the literature on the implementation context of eHealth interventions for caregivers of people with dementia. Municipality officials’ views indicated which implementation determinants they expected would influence the adoption, dissemination, and future implementation of eHealth interventions for caregivers of people with dementia in a municipal context. These insights were applied to tailored implementation strategies to facilitate the future implementation of interventions such as Myinlife and Partner in Balance.

## Introduction

### Electronic Health and Dementia Caregiving

Informal caregivers provide essential care to people with dementia, and this can have both positive [1] and negative effects on the caregivers’ daily lives [2-4]. Previous research has shown that these positive effects can include an enriched relationship with the person with dementia, whereas the negative effects include burnout and social isolation. Electronic health (eHealth) interventions are “*treatments, typically behaviorally based, that are operationalized and transformed for delivery via the Internet*” [5]. eHealth interventions for caregivers of people with dementia have shown evidence of effectiveness at improving a wide range of negative outcomes for these caregivers, including the reduction of depressive symptoms, anxiety, and burden [6-9]. In addition to the evidence of their effectiveness for caregivers, eHealth interventions have the potential to meet the challenges faced by many modern health care systems as result of aging populations and declining birth rates [10]. For instance, eHealth interventions can provide a lower threshold to participation, more opportunities for personalization, instant delivery, real-time feedback, and increased accessibility for reaching more isolated populations who experience difficulties in gaining access to traditional services [11,12].

However, very few psychosocial interventions for caregivers of dementia find their way from effectiveness trial to practice [13], including eHealth interventions for caregivers of people with dementia [14]. Bringing these evidence-based interventions into practice would be beneficial in a number of ways, including a more efficient allocation of research resources, a reduction of unnecessary research replication, and their eventual benefit to caregivers through sustainable implementation. Previous research has pointed toward the absence of knowledge on the contextual environment as a significant barrier for health system planners and implementers in translating these interventions into practice [15,16]. For instance, as eHealth interventions bypass the traditional delivery methods and care structures, many health care professionals and governing bodies do not know how to implement the interventions and modify existing structures and norms to incorporate them [17]. An important reason for this absence of knowledge on contextual factors is the gold standard of randomized controlled trials (RCTs) as evidence, which often lack crucial, qualitative implementation data [18]. There has been a call for more realistic, efficient research designs that take the context of the eHealth intervention into account [19]. For eHealth, this involves gaining insight into the relevant aspects and actors of organizations and communities in the real-life contexts where the interventions will be implemented. An example of such an implementation context is municipalities looking to offer Web-based support to caregivers of people with dementia.

### Study Aims

The aims of this study were twofold. First, this study aimed to gain insight into the views of municipality officials on the upcoming implementation of two eHealth interventions in their communities, to shed light on their reasons for adopting the technology and their strategies for dissemination and implementation. The two studied interventions were Myinlife, a Web-based platform to organize dementia care, and Partner in Balance, a Web-based course (see Methods). This study’s findings will help identify potential implementation determinants and fill the knowledge gap in the environmental and contextual factors that influence sustainable eHealth adoption, dissemination, and implementation. Second, this study aimed to translate the insights from these interviews into implementation strategies, to aid researchers in implementing evidence-based eHealth for dementia caregivers. The definitions for these terms as employed in this study are “*the decision of an organization or a community to commit to and initiate an evidence-based intervention*” for adoption, “*the active approach of spreading evidence-based interventions to the target audience via determined channels using planned strategies*” for dissemination, and “*the process of putting to use or integrating evidence-based interventions within a setting*” for implementation [20]. 

## Methods

### Study Setting

This study took place in the context of the euPrevent Senior-Friendly Communities (SFC) project [21], involving 32 municipalities from the Euregion Meuse-Rhine. Here, a municipality refers to a town or district that has a local government. Municipalities’ governing functions differ between countries, though in general they are responsible for local services that can include health care, education, recreation, and sport. This project ran from September 2016 to December 2019 and was implemented in the Euregion Meuse-Rhine, a border region covering parts of Belgium, Germany, and the Netherlands, which contains 150 municipalities. A total of 32 municipalities signed up to take part in the broader SFC project on a *first come, first serve* basis. The project first made an inventory of how the communities were already supporting their aging population and what they could still improve in this regard [22]. Afterward, municipalities chose activities from a so-called “activity buffet” consisting of 15 pre-existing activities. These activities addressed the mental health of older people, paying particular attention to dementia and age-related depression, including cultural activities such as a theatre production, a photo exhibition, consultations with experts on various topics, educational sessions on relevant topics and psychoeducation, creation, and organization of local social networks of elderly, and outreach activities. They also included two eHealth interventions to support caregivers of people with dementia: “Partner in Balance” and “Myinlife.” On average, each municipality chose to implement four activities.

### Studied Interventions

#### Partner in Balance

Partner in Balance is a blended care, 8-week, self-management intervention that helps caregivers of people with dementia adapt to their new roles. Detailed information about the program components and development is presented elsewhere [23]. In short, the blended care self-management program Partner in Balance consists of (1) a face-to-face intake session with a personal coach to familiarize participants with the program, choose Web-based modules, and set goals; (2) tailored Web-based thematic modules, including psychoeducation, behavioral modeling, reflective assignments, change plans, and email feedback from the coach over 8 weeks; and (3) a face-to-face evaluation session with the coach evaluating previously set goals. The coaches are health care professionals with experience in dementia care (eg, in the Netherlands, the Partner in Balance coaches are often dementia case managers). In a recent RCT, Partner in Balance was shown to be effective in improving caregivers’ sense of competency, self-efficacy, and quality of life [23,24].

#### Myinlife

Myinlife is a Web-based platform for caregivers of people with dementia to involve their social network in organizing care and share positive caregiving moments. In the Netherlands, Myinlife has been integrated into the Alzheimer Netherlands website. Myinlife has the potential to simplify caregiving and provide caregivers with more control over their agendas [25,26]. The platform consists of the following functionalities: profile, circles, timeline, calendar, helping, personal messages, care book, and compass.

### Study Design

In total, 9 of the 32 SFC municipalities opted to implement one of the two available eHealth interventions for caregivers of people with dementia in their communities: 6 municipalities chose Partner in Balance (4 in the Netherlands, 1 in Belgium, and 1 in Germany), whereas 3 chose Myinlife (2 in Belgium and 1 in Germany). The method of semistructured interviews was chosen because of its suitability to small-scale and flexible research, which matched the setting of this implementation study [27]. In each participating municipality, an open-ended, semistructured interview was conducted with the municipality official responsible for the implementation of the intervention. The interviews were on average 18.79 min long and took place in the period of about 6 months between the municipalities’ decision to adopt the interventions and their actual implementation. The interview questions were about the municipality official’s background, expectations concerning the implementation of the intervention, recommended strategies, and thoughts on eHealth in general. The complete interview guide can be found in Multimedia Appendix 1.

### Participants

In total, 8 in-person interviews were conducted. A ninth municipality chose to discontinue the implementation and delivered written answers to the interview. The reasons for this are discussed in the Results section. The officials interviewed in the remaining 8 participating municipalities had varying job descriptions. The majority described themselves as municipality policy officials, whereas some described themselves as employees responsible for specific activities concerning seniors, volunteers, demography, or specific local care facilities. Table 1 lists some specific characteristics of the 9 communities who had chosen Myinlife or Partner in Balance from the activity buffet. As the participating municipalities wished to remain anonymous, any identifying information has been left out.

**Table 1:** Characteristics of the participating municipalities.

Characteristics	Values, n
Number of municipalities that chose Partner in Balance	6
Number of municipalities that chose Myinlife	3
Municipality average general population^a^	36,376
Municipality average population aged >65 years^a^	7349
Municipality average estimated dementia population^a^	1434

^a^Population statistics sourced from the euPrevent Senior-Friendly Communities project [21,28].

### Data Collection

Ethical approval for the study was granted by Maastricht University’s Medical Ethical Oversight Commission under approval number 2018-0489. The 8 in-person interviews were conducted by one of the authors (HC) at each municipality’s town hall or equivalent between July 2018 and December 2018. Each participant received an information sheet about the background and aims of the study, in addition to information on how their data would be processed and stored. Each participant agreed to and signed an informed consent form. Interviews were conducted using a semistructured interview guide. Five interviews were conducted in Dutch, one in English, one in French, and one in German. The municipality that discontinued the implementation delivered written answers to the interview questions via email in Dutch.

### Data Analysis

The interviews were transcribed verbatim using transcription tool F5 (dr. dresing & pehl GmbH) . If conducted in a different language, transcriptions were translated into Dutch by two authors (HC and MS). The method of inductive analysis was chosen to explore the current perspectives of municipality officials, as this domain has not been much researched, and there was little notion of the factors and themes that might emerge [29,30]. On the basis of the inductive analysis with no pre-existing categories or themes, individual codes were grouped into themes and categories. Afterward, the themes and categories were compared in a consensus meeting with another author (MD) to resolve any differences of opinion, resulting in the final thematic analysis. Thus, this method of inductive analysis served to inform the study’s two objectives, which are explored using this study’s results, previous findings, and relevant literature in the Discussion. Two authors (HC and MS) independently coded the interviews using the described inductive thematic analysis method and software tool Atlas.ti for Macintosh (Atlas.ti Scientific Software Development GmbH).

## Results

### Overview

Four main themes emerged from the inductive thematic analysis: the eHealth intervention, the users, the organization, and the wider context. Within the themes, categories and groups were formed (Table 2). These themes can be seen as concentric circles, where the constructs in each widening circle are further removed from the smallest circle. The circles all interact with and influence each other. For the purposes of clarity and as a reflection of the chronological process, the following sections will start by discussing the outermost circle (the wider context) and then work inward toward the innermost circles (the organization, the users, and the eHealth intervention).

**Table 2:** Interview themes and categories.

Theme	Categories and subcategories
1. Wider context	1.1 Municipality’s context and political climate 1.2 Bottom-up versus top-down push for eHealth^a^ 1.3 Municipality values 1.3.1. Staying close to the citizen 1.3.2 Sustainability 1.3.3 Valuing volunteers 1.4 Societal factors 1.4.1 Self-management in health care 1.4.2 Sustainable integration with daily practice 1.4.3 Brand value 1.4.4 Increased needs for dementia care 1.4.5 Political support for digital future
2. Organization	2.1 Internal: The municipality 2.1.1 Implementation strategies 2.1.2 Attitudes 2.2 External: Collaboration with local organizations 2.2.1 Emphasize added value to external organization 2.2.2 Improving quality of care 2.2.3 Financial sustainability planning
3. Users	3.1 Caregivers 3.1.1 Dissemination: Through media, convincing through personal contact, gaining attention, events 3.1.2 Involving users 3.1.3 Personalization 3.1.4 Involvement in the implementation 3.2 Coaches 3.2.1 Difficult to find/train/guide coaches 3.2.2 Resource shortage 3.3 Lack of users’ digital abilities 3.3.1 Caregivers 3.3.2 Coaches
4. Intervention	4.1 Thoughts on eHealth 4.1.1 Must keep modules up to date 4.1.2 The Netherlands and Scandinavia at the forefront 4.1.3 Risks around data leaks 4.1.4 More familiarity with data systems than with apps 4.1.5 Easier to reach people than traditional interventions 4.2 Experiences with eHealth 4.2.1 As a database for patient information 4.2.2 In an educational context 4.2.3 In the media 4.2.4 No experience 4.3 Expectations about future success of intervention implementation 4.3.1 Ideal situation 4.3.2 Expectations

^a^eHealth: electronic health.

### Wider Context

The term wider context refers to the social, political, and economic settings in which the municipality resides. The results of the inductive thematic analysis indicated that the municipality officials viewed a number of social, political, and economic factors as contributors to the choice to adopt Partner in Balance and Myinlife. Examples of this include the increase in older people and dementia in the municipality, and the municipalities seeing the future as increasingly digital.

All over the community it’s the digital things that are successful and also the future and so, it would be strange if the medical part doesn’t take part.

Respondent 6

Furthermore, the fact that the intervention was evidence-based and had an academic “name brand recognition” resulting from its origins as a university research project, was a facilitating factor for some municipalities. Municipality officials mentioned that their choice of intervention depended on whether the intervention was in line with the values and policy of the municipality. In this regard, they mentioned that Myinlife and/or Partner in Balance matched their work on sustainability, caregiver support, and “staying close to the citizen.” When choosing which interventions to adopt for the project, the majority of municipalities reported having made the choice internally. However, two municipalities assembled a panel of lived-experience experts in dementia and caregiving and chose those activities which the panel identified as most relevant for their community. A final recurring theme regarding the choice to adopt the interventions was the bottom-up versus top-down approach to eHealth. Some respondents felt that eHealth is mainly pushed through top-down initiatives but that the population of their municipality does not express a desire for it.

Then you have the bottom-up or top-down approach, there is something to be said for the two of them. Now you started with the bottom-up, and yes, we are going to see how that goes and if that does not work, then we have to see if a top-down might become something. Then we have to see from which top we are going to start, so to speak.

Respondent 5

The reasons for adopting Myinlife and Partner in Balance seemed similar for both interventions. It is interesting to note that municipalities that had chosen to implement Partner in Balance emphasized both the advantages of the intervention for the caregiver as well as for the coach.

Besides adoption, the wider context also played a role in planning the upcoming dissemination and implementation of the interventions in the communities. For instance, politically, imminent elections and the merging of three municipalities into one municipality made concrete planning difficult, as the budget and officials responsible might change.

### Organization

When mapping the organizations involved in implementing Myinlife and Partner in Balance, the organizations were divided into two groups: internal (the municipality) and external (all local organizations they wanted to involve in the implementation). Concerning the internal attitudes of the municipality employees on the upcoming implementation, it appeared that the more familiar they were with the intervention, the more enthusiastic they were. Several long- and short-term implementation strategies were identified, such as appointing a contact person responsible for the intervention in the municipality, frequently checking up on and facilitating the intervention, and having a clear time plan.

It’s not like it’s ready-made. It’s still about people, you have to remember that, you have to facilitate that, you have to motivate that. If you don’t do that...everything depends on it, especially in this kind of work. If you think: Yes, now...I have thought it up nicely and it will come naturally...that will not work.

Respondent 2

Concerning the external cooperation with local organizations, the responses showed that municipalities felt it was particularly important that the eHealth intervention should improve health care in their community. In particular, they hoped it would connect various links in the local care network. Examples of organizations the municipalities wished to collaborate with for the upcoming implementation were local care homes, case management organizations, geriatric departments of hospitals, caregivers’ associations and support groups, general practitioners and other clinical professionals’ practices, social work, dementia expertise centers, and home care organizations. The municipality officials expressed some wariness toward the Web-based aspect of the interventions and emphasized that the interventions would only be useful if there were demonstrable improvement in local health care services, although they noted that this would be hard to measure. This described external involvement of local organizations can also be seen as a kind of implementation strategy, and it was mentioned in every interview. For Myinlife, the external cooperation mostly served the purpose of aid in advertising and publicizing to disseminate the intervention to the target users. For Partner in Balance, the external cooperation with local health care organizations was an essential part of recruiting the platform’s coaches, as they needed to have experience with both dementia and care.

But, yes, or that, will it make a difference later in care? When you talk about “care”- because that is central - I don’t know, does [Myinlife]contribute to increasing the quality of care?

Respondent 4

The respondents also foresaw significant barriers to implementation: Finding the time necessary to invest in publicizing and communicating about the intervention; finding coaches for Partner in Balance; convincing the older population of the platforms’ advantages; and financially guaranteeing the sustainability of the interventions. The municipality that discontinued the implementation and subsequently submitted answers to the questions by email chose to focus on this topic. This municipality felt that the inability of Partner in Balance to guarantee what a license would cost after the project’s end was a significant barrier. They also said the following:

There were too many unclear circumstances. Our neighbourhood teams had already started, the cooperating partners had full agendas and it was not clear what the costs were after the project.

Respondent 9

### Users

The theme *users* groups all statements from the municipality officials regarding who would be using the interventions. On the basis of their responses, two user groups were identified: the caregivers themselves and the coaches. The user group of the coaches is specific to Partner in Balance and does not apply to Myinlife. This finding of the coaches as a user group was interesting, as it had been expected that the coaches would be seen more as a part of the implementing staff described in theme category 3.2 (Table 2). However, it appeared that both the caregivers and the coaches were seen as target users of the platform by the community officials, both of whom required recruitment with specific dissemination strategies.

So finding the coaches of course and maybe...finding the coaches is of course natural, but it is a real challenge...And, of course, reaching sufficient informal caregivers who want to sign up for this.

Respondent 4

Concerning the recruitment of caregivers, municipality officials recommended focusing on younger caregivers, such as the children or grandchildren of people with dementia; involving local people with dementia and their caregivers in the implementation by consulting with them; being inclusive by trying to reach caregivers from all different backgrounds; and making sure the approach was personalized, as everyone has unique situations and needs. Specific dissemination strategies included media attention through both social media and press conferences, convincing local groups of the advantages of participation, and organizing face-to-face events. In this regard, the municipalities thought maintaining human contact was an essential part of the dissemination strategy. They proposed organizing stakeholder and caregiver meetings, rather than relying on digital and print communication.

I think, if we are going to focus purely on the partners of people with dementia, that we are only going to be able to reach very few people effectively. Because with a biased prejudice, maybe I am wrong, but I have this idea that older people are less open to web-based assistance than the younger generation. But I also know that there are many children who care for their mother or father with dementia, and we can reach them and if they have that knowledge they can hopefully also pass it on to the partner, so that we can also reach them directly. But I think that the online data is a difficult one. Plus, yes, it is now a one-off initiative - it has to be supported from [higher-up], and that must also remain on the agenda...

Respondent 5

Concerning the recruitment of coaches, again, municipality officials stressed a lack of resources on the coaches’ side, such as time and money, as a foreseeable barrier to effective dissemination and subsequently, implementation. As described in 3.2 (Table 2), most municipalities were keen to recruit both professionals and volunteers from local care organizations. However, one municipality also wanted to offer caregivers of people with dementia the chance to be coaches for Partner in Balance. They emphasized that is was important that these prospective lived-experience coaches would also be supported by a local dementia association. Furthermore, municipalities very often thought that both the caregivers and coaches of the target group would have a hard time with the Web-based aspect of the eHealth interventions.

Yes, most are actually received positively. The only thing is, we don’t know how many people are going to respond, so is that going to take off? (laughing) That is also, a, a consideration, that you sometimes hear, that I have heard a few times. But there is enough interest for that kind of stuff? You will only know that by trying and making it known and then seeing how much response there is.

Respondent 5

### Intervention

This theme describes the municipality officials’ thoughts on both the chosen platforms specifically and on the idea of eHealth in general. Though they did expect the Web-based aspect of the interventions to be a complicating factor, there were predominantly positive attitudes toward eHealth. However, most had not yet worked with eHealth themselves and had only heard about it. Of those that did have experience with eHealth, it was common that they had come into contact with it in an educational context, such as at a university or in a training workshop. Respondents were, in general, more familiar with eHealth in the context of online databases for patient information than with apps. Taking into account the limited sample size, there were no obvious relationships between the age or job description of the participants and their experiences with eHealth. Most respondents were optimistic about the chances of successfully implementing the intervention in their communities, but some also felt that it would not be suitable for everyone, or that it could only be really successful in the future (but not right now). When asked what the ideal implementation of Myinlife or Partner in Balance in their communities would look like 2 years from now, municipality officials said they would like to see it be an integrated part of local care services. Some also gave indications of the minimum number of users they would like to be on the platforms. These were quite small, the largest number being 30.

Well, ideal for me would be that it is well known, that it is completely embedded in the guidance of caregivers. That it is well-known to everyone who is confronted with dementia, that you can also get support from it as an informal caregiver, in addition to the regular care of course, the most optimal care for the person with dementia themselves. I think that's important. And that we have enough coaches, who are motivated to do this motivated and who...experience this as a meaningful activity.

Respondent 3

## Discussion

### Principal Findings

This study examined municipality officials’ views on the adoption, dissemination, and implementation of evidence-based eHealth interventions for caregivers of people with dementia in their local communities. The resulting inductive themes provided interesting insights that helped meet this study’s two objectives. First, these findings help fill the gap in the literature concerning the organizational and contextual factors that influence this process by identifying potential implementation determinants. Second, these findings aid the future implementation of eHealth interventions such as Myinlife and Partner in Balance by using these insights to formulate specific implementation strategies.

### Mapping the Implementation Context and Identifying Potential Determinants

Regarding the first, more general objective of mapping the implementation context to identify determinants that influence implementation, the following lessons were learned. The first lesson concerns the level of enthusiasm, both from the municipality officials and the target groups. The interviews demonstrated that municipalities were enthusiastic about the idea of implementing eHealth to support caregivers of people with dementia in their communities. Indeed, nine out of 32 municipalities in the Euregion chose to adopt and implement the two eHealth interventions on offer in the activity buffet. Previous research has explored the views of stakeholders concerning the implementation of health technologies, including care professionals, managers within home care or social work organizations, technology designers, and policy makers [31,32]. However, to our knowledge, none have explored the views of municipality officials. Knowing that municipalities are enthusiastic about these interventions is important for future developers looking for a viable implementation environment for their interventions. For instance, municipalities in the Netherlands are responsible for supporting their local caregivers and have funds allocated for this [33]. As the municipalities seem to have positive attitudes toward eHealth, as well as available funds and incentives to support caregivers, implementing eHealth through municipalities seems to be a viable option, especially if they focus on caregiver support.

Belgian and German municipalities are not necessarily responsible for municipal caregiver support, although they do facilitate care support through collaboration with local organizations and health care providers [21]. It is, however, important to note that the municipalities did mention experiencing a top-down push for eHealth and doubted whether their current older population would have an interest in using these interventions. Research into older adults’ attitudes toward eHealth interventions has shown mixed results [34-36], with evidence suggesting that older adults living in more rural areas (such as many of those included in this study) express less interest and capacity to use eHealth [37]. However, studies have also shown positive attitudes toward the use of eHealth both in older populations [38] and for younger caregivers [39]. Previous eHealth research has also mentioned enthusiasm from both target groups as well as the implementing organizations as an important implementation determinant [40].

The interviews demonstrated that, despite the Web-based and remote nature of eHealth interventions, the municipality officials all emphasized the importance of organizing face-to-face meetings with stakeholders and prospective users to facilitate a successful implementation. This builds on the findings from previously conducted Myinlife pilot studies, RCTs, and process evaluations, which showed a lack of effects on the trial’s quantitative outcomes [25,41]. In particular, the process evaluation [26] provided qualitative insights that led to continued implementation of Myinlife, such as the overwhelmingly positive user experiences. For instance, the Myinlife process evaluation emphasized that online and offline support was necessary to facilitate the caregivers’ knowledge of their own social support needs and available social capital. This is in line with municipality official’s views in this study, as they often mentioned the desire to organize meetings with the local caregivers. Future implementers should take into account that using events to promote the intervention and engage the target audience is recommended, especially for this older population, who might be harder to reach through online dissemination channels such as social media [42]. In addition, when comparing the concentric circles of influencing factors described here and in the Myinlife process evaluation [26], it is important to note that there is no circle discussing the influence of organizational factors in the Myinlife process evaluation. As is the case with many process evaluations, this is because of the fact that the process evaluation took place in a trial context, and there was no “external” implementation, as the implementation was carried out by the research team. However, it is important for researchers to consider these “internal” organizational factors in the process evaluation as well to facilitate the following implementation steps [14]. This need for more detailed information on the offline implementation aspect has been discussed in previous research [43] and would provide future implementers with useful information to make decisions regarding the viability of the intervention in its organizational context.

Next, the interviews also demonstrated that the municipalities considered the targeted recruitment of not only the caregivers but also of the coaches as an important contributor to successful implementation. Previously, the Partner in Balance process evaluation [44] had highlighted the importance of tailoring interventions to user characteristics and needs as well as the need for more research on the implementation process and context. Although the process evaluation did recommend an active role for health care professionals in guiding caregivers through the caregiving process, researchers had previously not considered the Partner in Balance coaches to be a part of the “user group.” They had instead seen them as a part of the implementing organization. This is contrasted by the findings from this study, where municipality officials saw both the caregivers and the coaches as two separate user groups that required specific recruitment strategies. Although disseminating the intervention to coaches using specific implementation strategies is resource intensive, there is evidence to show that the addition of this “blended” aspect to an eHealth intervention significantly enhances outcomes [7,45,46].

The uncertainty around how long the interventions would continue to be available after the project and how much they would cost was a significant barrier. Indeed, this issue caused one municipality to discontinue the implementation of Partner in Balance. The necessity of long-term business modeling to ensure sustainable implementation of eHealth interventions is in line with previous research, both for dementia [47] and other populations [48]. In this regard, mapping the surrounding health care context and other financial stakeholders in relation to the intervention characteristics is essential, for instance by applying the Business Model Canvas [49]. Insight in whether and how much municipalities would be willing to pay is essential to sustainably implement these interventions.

Importantly, the responses from the municipality officials show that the “name brand” (in this case, the name of Maastricht University and the Alzheimer Center Limburg) behind the eHealth intervention was an important factor in the decision to adopt the interventions. Not only the fact that they were evidence-based but also the fact that a reputable organization could vouch for the interventions was considered important. This is supported by previous research on health care provider adoption of eHealth [32] and emphasizes that developers of future interventions should consider highlighting the “name brand” value of their interventions, if applicable.

Finally, the process of conducting the qualitative, semistructured interviews with the municipalities was a very helpful exercise. These interviews helped avoid surprises in planning the later implementation by making expectations and agreements concrete. This fostered a sense of trust and understanding of the other parties’ needs. The interviews also allowed for the development of tailor-made implementation strategies, as recommended by Damschroder [50]. These tailor-made strategies also help provide a sense of ownership to the municipality, as they have a hand in designing them so that they fit the local context and stakeholders. Future eHealth developers looking to implement in municipalities or other organizations should consider holding similar “baseline interviews.”

### Translating Insights Into Specific Implementation Strategies

Regarding the second, more specific objective of formulating implementation plans for eHealth interventions such as Myinlife and Partner in Balance, based on the insights into municipality implementation determinants, the following strategies can be applied to aid researchers in their future implementation into practice:

1. *Regularly contacting municipality officials*: There will be one municipality official responsible for implementing the interventions in the municipality as an official contact person. It is important that the research team has regular contact with this person by having regular meetings to create goodwill and a productive rapport.
2. *Organizing face-to-face meetings with both local stakeholders and caregivers*: It is important to organize events to provide caregivers with information on caregiving and offering eHealth as a support tool. The municipalities’ wish to organize events to disseminate and promote the interventions further underscore this point that eHealth interventions, whatever their original design or intent, necessitate some amount of human contact and personal tailoring. Each community will organize a stakeholder meeting and a caregiver meeting to embed the interventions in the local, unique care landscape.
3. *Making use of existing local services*: Local dementia services in each municipality will be contacted to be part of the eHealth project teams, as well as help with the recruitment of both caregivers and coaches. In addition, other local services will be contacted including nursing and mental health care services, as well as youth groups, professional training and apprenticeship schools, and hospitals.
4. *Regular eHealth project meetings*: Each municipality will have an eHealth project team in addition to the municipality contact person. The contact person will be responsible for encouraging enthusiasm and increasing familiarity with the interventions and between team members. Members of the project team will include the municipality contact person, a representative from the research team, and the interested parties from the stakeholder and caregiver meetings.
5. *Promoting through online and offline campaigns*: In addition to the offline events, such as the stakeholder meetings, caregiver meetings, and eHealth project team meetings, municipalities will be encouraged to disseminate the interventions through any online channels they might have (such as websites, social media, and newsletters).
6. *Emphasizing name brand, evidence-based aspect*: All presentations and communication materials will emphasize the input of name-brand contributors, such as Maastricht University, Alzheimer Netherlands, ZonMW, the Alzheimer Center Limburg, INTERREG, and euPrevent.
7. *Collecting data to inform licensing model and ensure sustainability*: Describing the hours and financial resources needed during the project will help the municipalities decide whether the project will be sustainable in the future. These data will also help the research team and other future developers to budget for this need for continued, personalized support to the implementing organizations, informing sustainable business models and implementation plans. In this regard, it is important to consult with a local health authority to learn where their outcome priorities lie, so this can inform which data are collected.
8. *Tailoring more general strategies*: Each municipality’s implementation plan also includes strategies specific to the local population and services, such as collaborations with local technology companies and recruitment of local experts-by-experience as coaches. Given the finding that the health care and municipality context varies widely between countries, and even regions, certain aspects of the more general strategies will have to be tailored to the differing local services. For example, the Public Centers for Societal Welfare (*Openbaar Centrum voor Maatschappelijk Welzijn*) in Belgium are organized very differently and have different goals than the Dutch municipalities’ Law for Societal Support (*Wet Maatschappelijke Ondersteuning*) services.

The proposed strategies can help researchers in two ways. First, based on the experiences of this project, the strategies could help future researchers achieve a more successful collaboration with implementing organizations outside of the academic trial context. Second, applying these strategies could result in more much-needed data on the dementia eHealth implementation context, which many stakeholders (such as health insurers) claim is necessary for the scaling-up of these interventions. More generally, increasing the rate of successful, sustainable implementation of evidence-based eHealth interventions for caregivers of people with dementia can have significant societal advantages, including more targeted and efficient research funding, the possibility for caregivers of people with dementia to gain access to the interventions developed for them, as well as the opportunity for health care systems to provide more targeted, cost-efficient, and evidence-based Web-based dementia support [42].

### Limitations

To our knowledge, this is the first study that explores the views of municipality officials on implementing eHealth interventions in their local communities. However, this study does have a few important limitations. First, with the exception of the municipality that chose to discontinue the implementation and submitted the answers to the interview questions by email, all of the participating municipalities had already chosen to implement eHealth in their communities. This results in the study’s sample being biased to look favorably on eHealth implementation, as it does not take into account the views of those municipalities that did not choose these interventions. Furthermore, it is important to consider that this study interviewed municipalities that had signed up to be a part of the SFC project, and thus, could have been more motivated to successfully implement the interventions than “independent” municipalities might have been. Moreover, the SFC context limited the number of studied municipalities to those that had signed up to implement Partner in Balance and Myinlife, which resulted in a relatively small sample size and made it difficult to assess whether data saturation had been reached. Nevertheless, this study provides a useful overview of why the municipalities that opted to adopt these eHealth interventions did so, and many common themes were observed in the interviews. Second, as some of the authors were involved with the research institute that had developed both interventions and were responsible for their implementation, it is possible that the respondents were influenced to provide socially desirable responses. However, doubts and concerns were also expressed, and one municipality withdrew from the implementation, so there is reason to believe the municipalities still provided a nuanced and truthful account of their views. In addition, the researchers had no advantage associated with municipalities choosing one eHealth intervention over the other, or instead of the other SFC activities. Finally, it is important to remember that all implementation plans were hypothetical at the time of interviewing, as they had not yet started implementing the interventions. Although this approach made it possible to offer tailored implementation strategies, it also presumably made it difficult for the respondents to provide insight based on their experiences with the two specific eHealth interventions, although they did discuss their views on eHealth in general (Table 2, theme 4.2). Future research will evaluate the effectiveness of the proposed strategies.

### Conclusions

This study helps fill the gap in the literature concerning the implementation context of eHealth interventions for caregivers of people with dementia. The interviews provided information on how municipality officials view eHealth for caregivers of people with dementia and what they see as determinants of successful implementation. Proposed municipality implementation determinants included the enthusiasm from municipality officials to implement these interventions (despite a top-down push for them), the importance of face-to-face interviews in developing tailor-made implementation plans, regular face-to-face meetings with an eHealth project team, long-term sustainability planning by collecting data on required resources and benefits, and the facilitating effect of name brand recognition in adoption. Future research should collect data to inform pricing models to ensure long-term sustainability as well as evaluate the efficacy of the various proposed implementation strategies.

## Appendix

Multimedia Appendix 1 Interview guide for the semistructured qualitative interviews.

## Abbreviations

eHealth: electronic health
RCT: randomized controlled trial
SFC: Senior Friendly Communities
