# Supplementary material for: Perspectives From Municipality Officials on the Adoption, Dissemination, and Implementation of Electronic Health Interventions to Support Caregivers of People With Dementia: Inductive Thematic Analysis
Source: JMIR Aging. 2020 May 13;3(1):e17255. doi: 10.2196/17255 (PMC7254285; doi:10.2196/17255)
Supplement: Multimedia Appendix 1 [file aging_v3i1e17255_app1.docx]

**Appendix 1**

*Interview guide for the semi-structured qualitative interviews.*

1. What is your job description within this community?
2. How did you come to be responsible for this intervention?
3. Who else is involved in implementing this intervention?
4. Do you have any experience with eHealth, dementia, caregiving or implementation of change in general?
5. Yes = can you tell me something about that experience?
6. Could you please describe how you intend to implement this intervention in the community? (recruitment, stakeholder meeting, caregiver meeting, organizational decisions, staff, …). If so, do you have recommendations on how to do this?
7. How can we best reach the caregivers in this community?
8. Why/how did you choose [intervention]? What needs will this intervention meet that your community has?
9. What challenges do you foresee in implementing this intervention?
10. Is there enthusiasm in the municipality for this intervention:
    1. From the municipality itself?
    2. From the community?
11. Do you think caregivers will like and be helped by this intervention?
12. Where would you like to see this intervention in your community two years from now?
13. Where did you first hear about eHealth?
14. Do you have any examples of this? (other interventions, other names,…)
15. Do you think eHealth has the potential to be successful in your community?
16. So the main points I take away from this interview are [summary]. I appreciate the time you took for this interview. Is there anything else you think would be helpful for me to know?
